# Relationships among the Degree of Participation in Physical Activity, Self-Concept Clarity, and COVID-19 Stress in Adolescents

**DOI:** 10.3390/healthcare9040482

**Published:** 2021-04-19

**Authors:** Dae-Jung Lee

**Affiliations:** Department of Physical Education, Jeonbuk National University, Jeonju-si 54896, Korea; dleownd23@hanmail.net; Tel.: +82-41-270-2849; Fax: +82-82-41-270-2850

**Keywords:** adolescent, COVID stress, physical activity, self-concept clarity, structural equation modeling

## Abstract

The COVID-19 pandemic situation threatens the health of people globally, especially adolescents facing mental problems such as depression, anxiety, and obsessive-compulsive disorder due to constant COVID-19 stress. The present study aimed to provide basic data highlighting the need to alleviate COVID-19 stress among adolescents by promoting physical activity participation and strengthening self-concept clarity (SCC). To examine the relationships among participation in physical activity, SCC, and COVID-19 stress in pandemic-like conditions, the study was conducted on middle and high school students aged 14 to 19 and an online survey was conducted on 1046 Korean adolescents (521 male and 525 female students in the preliminary survey and main survey). Frequency, reliability, confirmatory factor, descriptive, and path analyses were performed using SPSS and AMOS 18.0. Participation in physical activity exerted a positive effect on SCC (*p* < 0.001) as well as a negative effect on COVID-19 stress (*p* = 0.031). Our findings also indicated that SCC exerted a negative effect on COVID-19 stress (*p* < 0.001). Regular participation in physical activity and strong SCC are also fundamental elements for alleviating COVID-19 stress. Given these results, state and local governments and educational institutions should encourage youth to participate in sports by suggesting policies, providing guidelines, and offering education. Such information may allow adolescents to endure and overcome COVID-19 stress during this critical period of life.

## 1. Introduction

The World Health Organization (WHO) declared a Public Health Emergency of International Concern on 30 January 2020 due to the worldwide spread of COVID-19 [1]. Many public health measures have been taken to slow the spread of the virus, such as developing treatment options, physical distancing, self-quarantine, and hand washing, and governments globally are containing the outbreak through border controls, containment, and contact tracing [2]. However, as of December 2020, more than 79 million confirmed cases and 1.7 million deaths had occurred worldwide [3]. In addition to its effects on physical health, the COVID-19 pandemic continues to exert unique and profound effects on mental health [4]. According to one study, more than 25% of the population have experienced severe stress or anxiety-related symptoms in response to COVID-19 [5].

In particular, adolescents have been faced with mental health problems, such as depression, anxiety, and obsessive-compulsive disorder due to constant stress from COVID-19 [4,6], and the suicide rate among adolescents is increasing [7]. The combination of hormonal changes during adolescence and multiple social stressors is not surprising considering that early adolescents are increasingly vulnerable to broad mood swings, emotional instability, and impairments in impulse control [8]. Accordingly, several studies have investigated the influence of the COVID-19 pandemic on stress among adolescents [9], and Taylor et al. [10] have developed a specific scale for assessing COVID-19 stress.

Stress is experienced throughout the life cycle [11] but can be relieved by physical activity, especially among adolescents [12,13] as well as adults [14]. Stress is related to self-concept clarity (SCC), which refers to the degree to which beliefs about oneself are clearly defined, internally consistent, and stable [15,16]. SCC is positively related to self-esteem, experiences of positive emotions, and extroversion, while it is negatively correlated with experiences of depression, anxiety, and negative emotions [16]. Treadgold [17] further reported that SCC exhibits a negative relationship with stress, suggesting that strong SCC can attenuate stress. According to a study by Lee-Flynn [18], the mechanism by which the components of self-concept influence the stress process remains unclear; nonetheless, strong SCC helps individuals to behave more resiliently in stressful situations.

The abovementioned findings indicate that there is a positive correlation between participation in physical activity and SCC which, in turn, exerts an influence on the level of stress. However, previous studies have only verified partial relationships between physical activity and SCC [19] and between SCC and stress [18]. Given that limited research has been conducted, the overall structural relationship among physical activity participation, SCC, and stress remains to be determined. In addition, studies investigating the unique type of stress associated with the COVID-19 pandemic are insufficient, especially those involving adolescents. Such unique stress is amplified by difficulties participating in physical activities due to social distancing, restrictions on the use of indoor and outdoor sports facilities, conversion to online classes at schools, and restrictions on sports viewing. Given that adolescence represents an important period of growth during one’s lifetime, additional studies are required to verify the comprehensive relationship among physical activity, SCC, and COVID-19 stress in this population.

Therefore, in the present study, the relationships among participation in physical activity, SCC, and COVID-19 stress are empirically examined. For this purpose, the following hypotheses are proposed:

**Hypothesis** **1** **(H1).** *There are differences in SCC and COVID-19 stress according to gender, school level, and the frequency, time, and duration of participation in physical activity.*

**Hypothesis** **2** **(H2).** *Participation in physical activity positively affects SCC.*

**Hypothesis** **3** **(H3).** *Participation in physical activity negatively affects COVID-19 stress.*

**Hypothesis** **4** **(H4).** *SCC negatively affects COVID-19 stress.*

## 2. Materials and Methods

### 2.1. Participants

A total of 1046 adolescents from the Republic of Korea were selected for the present study using convenience sampling, which is a non-probabilistic sampling method. Specifically, 226 adolescents were selected for the preliminary survey and 820 adolescents were selected for the main survey. The selection of participants for the study included students from 12 schools (six middle schools and six high schools) in Jeollabuk-do province, Korea. To induce voluntary participation, the purpose of the study, method of the survey, time required for the survey, and benefits and losses from participating in the study were explained. Furthermore, the definition and type of physical activity were presented so that students could understand the purpose of the research in advance. Recruited students participated in an online survey after obtaining consent from their guardians. All respondents participated voluntarily. All the survey data of research subjects were used as research data because missing questionnaires were prevented from being returned by the nature of the online survey. No inclusion/exclusion criteria for potential participants were set. The demographic characteristics of participants are presented in Table 1. This study was conducted after obtaining ethical approval from the Institutional Review Board of Jeonbuk National University (JBNUIRB-2020-12-020-001).

### 2.2. Instruments

Demographic characteristics including gender, school level, presence or absence of contact with confirmed patients, and the frequency, time, and duration of physical activity participation were assessed. Participation in physical activity was defined based on the model of sports participation developed by Snyder [20], and items from a scale whose reliability and validity have been verified by Lee et al. [21] were used. Although Snyder [20] defined sports participation based on three subvariables (cognitive, behavioral, and affective participation), only three items related to behavioral participation were utilized given the purpose of our study. SCC was first described by Campbell et al. [16], and items from an SCC scale whose reliability and validity have been verified by Becht et al. [22] were utilized. SCC was thus assessed based on responses to items such as “My thoughts about myself often conflict with each other”, and “I spend a lot of time thinking about who I really am”. Questions 1, 2, 3, 4, 5, 7, 8, 9, 10, and 12 are reverse scored. COVID-19 stress was assessed by modifying and supplementing the COVID-19 Stress Scale developed by Taylor et al. [10]. The COVID-19 Stress Scale includes five questions related to danger and contamination, four questions related to socio-economic consequences, four questions related to xenophobia, four questions related to traumatic stress, and four questions related to compulsive behavior.

Participation in physical activity, SCC, and COVID-19 stress were rated using a five-point scale, as follows: “absolutely” = 5, “yes” = 4, “normal” = 3, “no” = 2, and “not at all” = 1. Scores closer to 5 were considered to indicate higher perception of the variable.

### 2.3. Reliability and Validity of the Scale

An exploratory factor analysis was conducted to check the validity of each variable. As this study used an already validated questionnaire from a prior study, the validity of each variable was further integrated and verified through a confirmatory factor analysis, the results of which are presented in Table 2.

In our analysis of the suitability of the proposed model, the following was observed: root mean square residual (RMR) = 0.068, normed fit index (NFI) = 0.806, incremental fit index (IFI) = 0.836, comparative fit index (CFI) = 0.835, and root mean square error of approximation (RMSEA) = 0.074. These findings indicated that the scale did not meet standard values. Therefore, based on the squared multiple correlation (SMC) value, items 6, 8, 11, and 12 of SCC were removed, as were the four items of the COVID-19 Stress Scale related to traumatic stress. A subsequent analysis revealed that the fit of the revised model was as follows: RMR = 0.079, NFI = 0.905, IFI = 0.918, CFI = 0.901, and RMSEA = 0.080—all of which are considered acceptable.

Next, the validity of the model was verified based on the results of the confirmatory factor analysis. First, three methods were utilized to verify convergent validity: standardized regression weights, average variance extracted (AVE), and construct reliability. The specific confirmatory factor analysis results are shown in Table 3. The range of standardized regression coefficients for all variables was 0.542–0.937, and the significance critical ratio (CR) was 1.965 or higher. In addition, the construct reliability (appropriate at 0.7 or more) ranged from 0.822 to 0.894, and the AVE (appropriate at 0.5) ranged from 0.514 to 0.626. Thus, convergent validity was secured by satisfying all three conditions.

Next, the correlation between the constituent concepts and the AVE was assessed to verify discriminant validity. The results of this analysis are shown in Table 4. Discriminant validity was verified by selecting the two variables with the highest correlation and comparing them with the value of the AVE. The square of the highest correlation coefficient for “SCC↔COVID-19 stress” was 0.320, which was lower than the AVE between SCC (0.514) and COVID-19 stress (0.541), indicating that the discriminant validity between the variables had been secured.

To verify the reliability of the scales used in this study, Cronbach’s α was utilized, which verifies the internal consistency of an item. The results of this analysis are shown in Table 5. Cronbach’s α for all observed variables ranged from 0.787 to 0.916, indicating that all values were above the standard value of 0.6 and therefore exhibited high internal consistency.

### 2.4. Procedure and Data Analysis

In this study, a preliminary survey of 226 adolescents in Jeollabuk-do province from 6 to 10 December 2020 was conducted. The main survey was conducted among 820 adolescents using the Naver Online Survey Form (http://naver.me/FT0RFrGe) from 28 December 2020 to 11 January 2021. Data were collected using an online questionnaire, the reliability and validity of which were verified. The obtained data were analyzed using SPSS 18.0 and AMOS 18.0 (IBM Corp., Armonk, NY, USA). First, a frequency analysis was conducted to confirm the general characteristics of the participants. Then, confirmatory factor analysis and Cronbach’s α were used to verify the validity and reliability of the scale, respectively. Independent samples t-test and one-way analyses of variance (ANOVA) were used to examine differences in each variable based on demographic characteristics. Lastly, path analysis was conducted to account for errors and verify the relationships among participation in physical activity, SCC, and COVID-19 stress more accurately. The statistical significance level was set at 0.05.

## 3. Results

### 3.1. Descriptive Analysis

Descriptive statistics for each of the variables (participation in physical activity, SCC, COVID-19 stress) according to factors and subfactors are presented in Table 6. The mean was distributed between 2.50 and 3.45, while the standard deviation was distributed between 0.78 and 1.22. Next, skewness and kurtosis were examined. In general, when skewness <±3.0 and kurtosis <±10.0, the distribution can be classified as normal [23,24]. Our analysis revealed that the absolute value of skewness was distributed from 0.009 to 0.444, while the absolute value of kurtosis was distributed from 0.058 to 1.195. Therefore, the normality of the structural equation was verified.

### 3.2. Differences between Groups for Each Variable

#### 3.2.1. Gender Differences in Each Variable

Differences in each variable according to gender are shown in Table 7. Male students (M = 3.15) exhibited significantly greater participation in physical activity than female students (M = 2.54). In addition, SCC was significantly stronger in male students (M = 3.20) than in female students (M = 3.00). Levels of COVID-19 stress variables such as danger and contamination, socio-economic consequences, xenophobia, and compulsivity were all significantly lower in male students than in female students.

#### 3.2.2. Differences in Each Variable According to School Level

Differences in each variable according to school level are shown in Table 8. There was no significant difference in physical activity participation between middle school students (M = 2.85) and high school students (M = 2.81). In addition, there were no significant differences in SCC between middle school students (M = 3.12) and high school students (M = 3.06). Although there were no differences in COVID-19 stress variables such as danger and contamination or xenophobia based on school level, perceptions of socio-economic consequences were significantly lower among high school students (M = 2.41) than among middle school students (M = 2.57). By contrast, compulsivity levels were significantly lower among middle school students (M = 2.45) than among high school students (M = 2.61).

#### 3.2.3. Differences in Each Variable According to Frequency of Physical Activity

Differences in each variable according to the frequency of physical activity participation per week are shown in Table 9. Participation in physical activity (F = 69.186, *p* < 0.001) and SCC (F = 16.127, *p* < 0.001) differed significantly based on the frequency of physical activity per week. Among the COVID-19 stress variables, there were significant differences in perceptions of danger and contamination (F = 14.838, *p* < 0.001) and xenophobia (F = 13.723, *p* < 0.001) based on the frequency of physical activity per week. However, no such differences were observed for socio-economic consequences (F= 0.766, *p* = 0.513) or compulsivity (F = 2.611, *p* = 0.050).

#### 3.2.4. Differences in Each Variable According to the Duration of Each Physical Activity Session

Differences in each variable according to the duration of each physical activity session are shown in Table 10. Participation in physical activity (F = 74.486, *p* < 0.001), SCC (F = 76.323, *p* < 0.001), danger and contamination (F = 35.315, *p* < 0.001), socio-economic consequences (F = 3.345, *p* = 0.019), xenophobia (F = 36.283, *p* < 0.001), and compulsivity (F = 12.752, *p* < 0.001) were significantly influenced by the duration of each physical activity session.

#### 3.2.5. Differences in Each Variable According to the Overall Duration of Participation in Physical Activity

Differences in each variable according to the duration of participation in physical activity in months are presented in Table 11. Physical activity participation (F = 86.210, *p* < 0.001) and SCC (F = 76.525, *p* < 0.001) were significantly influenced by the overall duration of participation in physical activity. In addition, the duration of physical activity in months significantly influenced the danger and contamination (F = 36.622, *p* < 0.001), xenophobia (F = 33.526, *p* < 0.001), and compulsivity (F = 15.562, *p* < 0.001) subdimensions of COVID-19 stress. However, no such effects were observed for socio-economic consequences (F = 1.104, *p* = 0.347).

### 3.3. Path Analysis

Three latent variables (participation in physical activity, SCC, and COVID-19 stress) and 15 observed variables (three for physical activity participation, eight for SCC, and eight for COVID-19 stress) were used to verify the hypothetical model. The fitness of the set model was verified before the path analysis was performed, and the results are listed in Table 12. The fit of the proposed model was evaluated as acceptable, with RMR = 0.079, NFI = 0.905, IFI = 0.918, CFI = 0.918, and RMSEA = 0.080.

**Figure 1:**
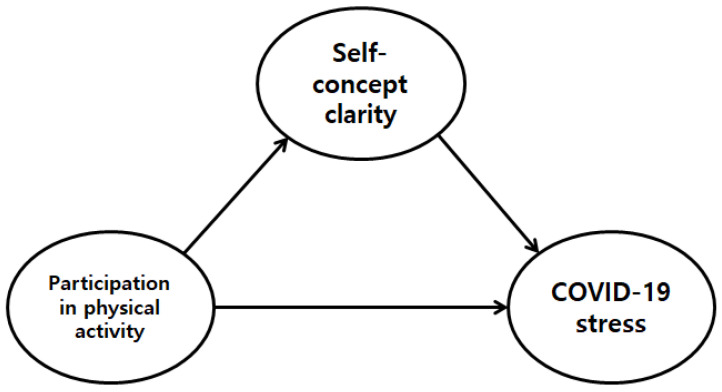
Path analysis model.

The verification results derived via the path analysis are shown in Figure 1 and Table 13. First, the path coefficient that verified the effect of physical activity participation on SCC was 0.152 (t = 3.856), which was statistically significant. This finding indicates that participation in physical activity exerted a positive effect on SCC. Second, the path coefficient that verified the effect of physical activity participation on COVID-19 stress was −0.078 (t = −2.152), which was also statistically significant. Thus, this result indicates that participation in physical activity exerted a negative effect on COVID-19 stress. Third, the path coefficient that verified the effect of SCC on COVID-19 stress was −0.555 (t = −12.032), which was again statistically significant, indicating that SCC exerted a negative effect on COVID-19 stress.

## 4. Discussion

In the present study, the relationships among participation in physical activity, SCC, and COVID-19 stress in an adolescent population were empirically examined. Our findings were as follows. First, there were partial differences in each variable according to demographic variables. Specifically, male students exhibited significantly higher rates of sports participation and significantly stronger SCC than female students. Furthermore, female students provided significantly poorer ratings for all subdimensions of COVID-19 stress, indicating that they were more vulnerable to such stress than male students in all subdimensions. Qi et al. [25] also reported that men exhibited significantly higher levels of physical activity than women during the pandemic. Furthermore, in their study of 1127 adolescents, You and Shin [26] reported that male adolescents exhibited significantly stronger SCC than female adolescents. Thus, our results are partially consistent with those of these two previous investigations. Shevlin et al. [27] further reported that women experience more anxiety, depression, and stress due to COVID-19, which is also in accordance with our findings. However, Qi et al. [25] reported no difference in COVID-19 stress levels between men and women. This discrepancy may reflect the fact that COVID-19 stress is not based on the inner characteristics of men and women, but that it is caused by changes in one’s surroundings and life associated with pandemic-like conditions. Taken together, these findings highlight the need to increase the level of physical activity among female students and build an environment that can alleviate stress by strengthening SCC. In addition, the results of Qi et al. [25] suggest that more educated people are more likely to have an effective strategy for collecting appropriate information to manage COVID-19 stress. Thus, information regarding how physical activity and SCC are related to COVID-19 stress should be provided in schools and via public broadcasting so that girls can learn the importance of each variable verified in this study.

Although school level did not appear to influence participation in physical activity or SCC, our analysis indicated that it had a significant effect on COVID-19 stress. In particular, no differences in physical activity participation between middle and high school students were observed, which is in contrast to the results of several previous studies [28,29]. Given that the school curriculum exerts a significant impact on levels of physical activity among adolescents [28], this finding may be explained by the absence of physical activities (gym time, sports club time) in the school curriculum and restrictions on the use of sports facilities due to COVID-19. Our results suggest that it is necessary to provide guidelines for physical activities that can be performed outdoors or at home individually when school-based physical activities cannot be implemented due to COVID-19.

The present analyses also revealed that higher levels of physical activity (frequency, time per session, and overall duration) were associated with greater participation in physical activity, stronger SCC, and lower COVID-19 stress. Qi et al. [25] reported a positive correlation between physical activity levels and health-related quality of life as well as a negative correlation between physical activity levels and COVID-19 stress. In addition, Rueggeberg et al. [30] stated that regular exercise and physical activity can promote life satisfaction and happiness in stressful situations. Taken together, these results indicate that SCC is strengthened and COVID-19 stress is alleviated through periodic and long-term participation in physical activity, rather than through brief and infrequent participation. Accordingly, measures should be taken to assist adolescents in reducing stress by promoting long-term, continuous participation in physical activity. The Department of Health and Human Services [31] recommends at least 30 min of moderate physical activity per day or at least 20 min of vigorous physical activity at least once every 2 days. To achieve such durations at school, outdoors, and at home, guidelines should be developed in consideration of a mid- to long-term perspective.

The present analysis further revealed that participation in physical activity exerted a significant positive effect on SCC. The development of SCC in adolescence is associated with relationships between oneself and parents or friends [32], self-esteem [33,34], and body image [35]. These variables are naturally derived from continuous participation in physical activities, and it is thought that regular participation in physical activities among adolescents strengthens SCC. For example, continuous participation in physical activity increases self-esteem by enhancing physical fitness awareness, inducing positive changes in body mass index, and improving body image [35,36]. The social aspects of participating in physical activity also naturally improve relationships with parents [37] and friends [38].

In addition, adolescents’ participation in physical activity exerted a significant negative effect on COVID-19 stress. These results are consistent with those of Carmack et al. [39], who demonstrated that exercise for health lowers the level of stress perception in stressful situations. Our findings are also partially consistent with those of Qin et al. [40], who indicated that people who participate in vigorous physical activity experience better emotional states, while those who do light physical activity exhibit the opposite tendency.

Research has demonstrated that levels of outdoor physical activity have decreased and that sedentary time has increased during the COVID-19 pandemic as a result of social distancing and lockdown [1]. In their study of 645 individuals, Qi et al. [25] reported that 64.8% of participants had low levels of physical activity during the pandemic, that their sedentary time increased, and that more than 50% of participants experienced moderate stress. If this trend persists in adolescents, it could lead to indirect physical and mental health problems as well as direct infection with COVID-19 [41]. Therefore, the need to provide professional guidelines for promoting exercise health remains essential. However, because intolerance to COVID-19 is related to eating disorders and compulsive exercise [42], obsession with exercise should be avoided in order to relieve the stress caused by COVID-19 and maintain health.

In the present study, SCC exerted a significant negative effect on COVID-19 stress among adolescents. SCC, an element of one’s own knowledge, allows individuals to respond more flexibly to stressful situations and respond better in social environments [15]. People with a clear self-concept have a consistent internal standard and concept of self, a clear identity, and are less dependent on external standards [43]. Conversely, according to Van Dijk et al. [32], the period of adolescence is associated with a less obvious self-concept, which can lead to higher levels of depression and anxiety. The results of the present and previous studies suggest that strong SCC enables one to respond appropriately to pandemic-related stressors. Therefore, to minimize COVID-19 stress, it is necessary to focus on strengthening SCC and promoting continuous participation in physical activity. Notably, when the epidemic crisis worsens, people in the affected area are more likely to panic, feel helpless, and fear getting sick [5]; therefore, it may be impossible to maintain a daily exercise or physical activity routine. In such situations, a focus on strengthening SCC may allow individuals to endure and overcome COVID-19 stress. For example, Van Dijk et al. [32] indicated that those who continued to talk about self-concept with their parents had strengthened SCC. Therefore, an environment in which self-concept-related dialogue can take place between parents and adolescents at home and between teachers and adolescents at school should be created when lockdown occurs during a pandemic.

## 5. Strengths, Limitations, and Future Studies

The limitations of the present study suggest the following future studies. First, the core aims of this study were to examine the relationship between participation in physical activity and SCC, and to present basic data that highlight the need to alleviate COVID-19 stress in adolescents. Our results indicated that regular participation in physical activity and high SCC can attenuate COVID-19 stress. However, excluding the preliminary study, 102 of 820 participants stated that there were confirmed cases of COVID-19 around them, representing only 12.4% of all participants. Therefore, for a more accurate analysis of the effects of regular participation in physical activity and high SCC on COVID-19 stress in adolescents, studies should involve adolescents living in regions with a high number of confirmed cases of COVID-19. Second, this study was able to derive meaningful results by setting physical activity participation, physical activity level, and SCC as variables that can affect COVID-19 stress. However, other variables may also influence COVID-19 stress among adolescents under pandemic conditions. For example, Lee-Flynn et al. [18] suggested that self-esteem affects stress in the short term and negatively affects stress by interacting with SCC in the long term. Therefore, a multidimensional analysis of variables that can affect COVID-19 stress is required. Third, all variables in this study were assessed using self-report measures. Given that self-report scales do not necessarily reflect actual behavior, questions regarding validity may be raised. Fourth, no other confounding variables that were assessed along with physical activity to determine the strength of the association with the responses compared with physical activity were investigated. Therefore, future studies should attempt to adopt more objective research methods to complement our subjective findings. The strength of this study is that it helped to shed light on the relationship between COVID-19 stress—which has recently become a public health issue—and participation in physical activity and SCC. Furthermore, as this study explores a large sample in the Republic of Korea, it should be possible to generalize its findings to other research settings.

## 6. Conclusions

In the present study, partial differences in each variable according to demographic characteristics were first observed. More importantly, our analysis revealed that participation in physical activity exerts a positive effect on SCC and a negative effect on COVID-19 stress. In addition, a negative effect of SCC on COVID-19 stress was also observed. Long-term, widespread, and unprecedented social distancing and isolation measures have led to negative psychological effects such as emotional disorders, depression, stress, low mood, irritation, panic attacks, phobia symptoms, insomnia, anger, emotional exhaustion, post-traumatic stress symptoms [41], and even suicide [44]. To protect the health of adolescents living in this situation, it is necessary to relieve the stress associated with pandemic-related conditions. Accordingly, the present study focused on the ability of physical activity participation and SCC to alleviate COVID-19 stress. Before COVID-19, physical activity and exercise focused on improving quality of life or preventing adult diseases, and SCC was regarded as important for self-development in general. Our findings indicate that regular participation in physical activity and strong SCC are also fundamental elements for alleviating COVID-19 stress. Given these results, state and local governments and educational institutions should encourage youth to participate in sports by suggesting policies, providing guidelines, and offering education.

For example, stretching exercises and gymnastics videos that could help promote physical activity throughout life should be produced and distributed, while guidelines for walking programs should be prepared to minimize the decrease in physical activity caused by the COVID-19 pandemic. In addition, to restore psychological trauma, it is necessary to promote youth market-style psychological recovery support programs as well as provide healing programs using cultural content at the region level.

## Figures and Tables

**Table 1:** Demographic characteristics of the participants.

Variables	Categories	Preliminary Study		Main Study	
		*n*	%	*n*	%
Gender	Male	124	54.9%	397	48.4%
	Female	102	55.1%	423	51.6%
School level	Middle school	132	58.4%	458	55.9%
	High school	94	51.6%	362	44.1%
Contact with confirmed patients	Presence	13	5.8%	102	12.4%
	None	213	94.2%	718	87.6%
Frequency of physical activity	None	104	46.0%	433	52.8%
	Once a week	42	18.6%	128	15.6%
	2–3 times a week	51	22.6%	144	17.6%
	4 times or more	29	12.8%	115	14.0%
Time spent performing physical activity per session	None	106	46.9%	404	49.3%
	Up to 30 min	61	27.0%	146	17.8%
	31–60 min	39	17.3%	138	16.8%
	60 min or more	20	8.8%	132	16.1%
Duration of participation in physical activity	None	105	46.5%	430	52.4%
	Up to 3 months	54	23.9%	168	20.5%
	3–6 months	38	16.8%	76	9.3%
	6 months or more	29	12.8%	146	17.8%
Total		226	100%	820	100%

**Table 2:** Goodness-of-fit in the confirmatory factor analysis.

Models	Root Mean Square Residual	Normed Fit Index	Incremental Fit Index	Comparative Fit Index	Root Mean Square Error of Approximation
Initial model	0.068	0.806	0.836	0.835	0.074
Final model	0.079	0.905	0.918	0.901	0.080

**Table 3:** Results of the confirmatory factor analysis.

Variables			Non-Standardized Coefficient	SE	CR	*p*	Standardized Coefficient	Standardized Coefficient	AVE
Participation in physical activity	→	Item 3	1.000	-	-	-	0.623	0.830	0.626
	→	Item 2	1.572	0.080	19.776	<0.001 ***	0.889		
	→	Item 1	1.630	0.084	19.490	<0.001 ***	0.937		
Self-concept clarity	→	Item 10	1.179	0.068	17.434	<0.001 ***	0.689	0.894	0.514
	→	Item 9	1.215	0.068	17.991	<0.001 ***	0.715		
	→	Item 7	1.155	0.061	19.019	<0.001 ***	0.763		
	→	Item 5	1.000	-	-		0.668		
	→	Item 4	1.148	0.063	18.102	<0.001 ***	0.720		
	→	Item 3	0.999	0.060	16.608	<0.001 ***	0.652		
	→	Item 2	1.090	0.057	19.148	<0.001 ***	0.770		
	→	Item 1	0.846	0.053	16.062	<0.001 ***	0.628		
COVID-19 Stress	→	Compulsivity	1.000	-	-	-	0.641	0.822	0.541
	→	Xenophobia	1.457	0.088	16.603	<0.001 ***	0.754		
	→	Socio-economic consequences	0.975	0.075	12.959	<0.001 ***	0.542		
	→	Danger and contamination	1.221	0.071	17.102	<0.001 ***	0.810		

SE = standard error, CR = critical ratio, AVE = average variance extracted, *** *p* < 0.001, tested via confirmatory factor analysis.

**Table 4:** Verification of discriminant validity.

Variables	Correlation between the Constituent Concepts			Average Variance Extracted
	Participation in Physical Activity	Self-Concept Clarity	COVID-19 Stress	
Participation in physical activity	1.000	-	-	0.626
Self-concept clarity	0.152 ***	1.000	-	0.514
COVID-19 stress	−0.162 ***	−0.566 ***	1.000	0.541

*** *p* < 0.001, tested via correlation analysis.

**Table 5:** Analysis of reliability.

Latent Variables		Cronbach’s α
Participation in physical activity		0.855
Self-concept clarity		0.884
COVID-19 stress	Danger and contamination	0.800
	Socio-economic consequences	0.912
	Xenophobia	0.916
	Compulsivity	0.787

**Table 6:** Descriptive analysis (full mark: 5.0).

Variables		Mean	Standard Deviation	Skewness	Kurtosis
Participation in physical activity	Item 1	2.81	1.20	−0.013	−1.014
	Item 2	2.83	1.22	−0.120	−1.195
	Item 3	2.87	1.10	0.208	−0.409
Self-concept clarity	Item 1	3.19	0.93	−0.218	0.121
	Item 2	2.97	0.98	−0.045	−0.469
	Item 3	3.00	1.06	−0.206	−0.619
	Item 4	2.82	1.10	0.037	−0.903
	Item 5	3.16	1.03	−0.297	−0.524
	Item 7	2.94	1.04	−0.060	−0.677
	Item 9	3.45	1.17	−0.190	−0.885
	Item 10	3.24	1.18	0.003	−0.794
COVID-19 stress	Danger and contamination	3.19	0.78	0.009	−0.058
	Socio-economic consequences	2.50	0.93	0.444	0.170
	Xenophobia	3.04	1.00	0.122	−0.599
	Compulsivity	2.52	0.81	0.421	0.337

**Table 7:** Gender differences in each variable.

	Sort	Mean ± Standard Deviation		*t*-Value	*p*
Variables		Male (*n* = 397)	Female (*n* = 423)		
Participation in physical activity		3.15 ± 0.98	2.54 ± 0.99	8.861	<0.001
Self-concept clarity		3.20 ± 0.81	3.00 ± 0.76	3.715	<0.001
Danger and contamination		3.02 ± 0.76	3.35 ± 0.77	−6.277	<0.001
Socio-economic consequences		2.43 ± 0.90	2.57 ± 0.96	−2.127	0.034
Xenophobia		2.86 ± 0.98	3.20 ± 0.99	−4.902	<0.001
Compulsivity		2.42 ± 0.78	2.61 ± 0.82	−3.515	<0.001

**Table 8:** Differences in each variable according to school level.

	Sort	Mean ± Standard Deviation		*t*-Value	*p*
Variables		Middle School (*n* = 458)	High School (*n* = 362)		
Participation in physical activity		2.85 ± 1.06	2.81 ± 1.00	0.542	0.588
Self-concept clarity		3.12 ± 0.81	3.06 ± 0.76	1.112	0.266
Danger and contamination		3.15 ± 0.79	3.23 ± 0.77	−1.409	0.159
Socio-economic consequences		2.57 ± 0.94	2.41 ± 0.92	2.382	0.017
Xenophobia		3.06 ± 1.01	3.01 ± 0.99	0.731	0.465
Compulsivity		2.45 ± 0.79	2.61 ± 0.83	−2.766	0.006

**Table 9:** Differences in each variable according to frequency of physical activity.

	Sort	Mean ± Standard Deviation				F	*p*	Post Hoc
Variables		A (*n* = 433)	B (*n* = 128)	C (*n* = 144)	D (*n* = 115)			
Participation in physical activity		2.43 ± 1.04	2.94 ± 0.85	3.29 ± 0.77	3.65 ± 0.67	69.186	<0.001	A < B < C < D
Self-concept clarity		2.93 ± 0.72	3.15 ± 0.71	3.28 ± 0.82	3.42 ± 0.93	16.127	<0.001	A < B, C, D
Danger and contamination		3.35 ± 0.77	3.14 ± 0.67	2.94 ± 0.79	2.96 ± 0.78	14.838	<0.001	A < C, D
Socio-economic consequences		2.51 ± 1.02	2.54 ± 0.88	2.55 ± 0.87	2.55 ± 0.87	0.766	0.513	-
Xenophobia		3.23 ± 0.99	2.99 ± 0.97	2.70 ± 0.99	2.70 ± 0.99	13.723	<0.001	A < B, C, D
Compulsivity		2.55 ± 0.84	2.62 ±0.67	2.48 ± 0.85	2.35 ± 0.75	2.611	0.050	-

A: None, B: Once a week, C: 2–3 times a week, D: 4 or more times a week.

**Table 10:** Differences in each variable according to the duration of each physical activity session.

	Sort	Mean ± Standard Deviation				F	*p*	Post Hoc
Variables		A (*n* = 404)	B (*n* = 146)	C (*n* = 138)	D (*n* = 132)			
Participation in physical activity		2.40 ± 1.03	2.90 ± 0.91	3.23 ± 0.87	3.66 ± 0.52	74.486	<0.001	A < B < C < D
Self-concept clarity		2.91 ± 0.70	2.88 ± 0.63	3.08 ± 0.78	3.93 ± 0.68	76.323	<0.001	A, B, C < D
Danger and contamination		3.37 ± 0.77	3.25 ± 0.73	3.14 ± 0.78	2.62 ± 0.60	35.315	<0.001	A < C < D, B < D
Socio-economic consequences		2.51 ± 1.04	2.67 ± 0.87	2.48 ± 0.91	2.32 ± 0.60	3.345	0.019	B < D
Xenophobia		3.24 ± 1.00	3.17 ± 0.86	3.03 ± 1.03	2.27 ± 0.74	36.283	<0.001	A, B, C < D
Compulsivity		2.57 ± 0.84	2.66 ± 0.79	2.58 ± 0.84	2.14 ± 0.52	12.752	<0.001	A, B, C < D

A: None, B: Up to 30 min, C: 30–60 min, D: More than 60 min.

**Table 11:** Differences in each variable according to the duration of participation in physical activity in months.

	Sort	Mean ± Standard Deviation				F	*p*	Post Hoc
Variables		A (*n* = 430)	B (*n* = 168)	C (*n* = 76)	D (*n* = 146)			
Participation in physical activity		2.39 ± 1.03	2.99 ± 0.86	3.40 ± 0.87	3.66 ± 0.47	86.210	<0.001	A < B < C, D
Self-concept clarity		2.91 ± 0.72	2.87 ± 0.54	3.15 ± 0.88	3.88 ± 0.71	76.525	<0.001	B < C < D, A < D
Danger and contamination		3.37 ± 0.78	3.27 ± 0.69	3.00 ± 0.76	2.66 ± 0.63	36.622	<0.001	A < C < D, B < D
Socio-economic consequences		2.51 ± 1.04	2.58 ± 0.86	2.47 ± 0.92	2.39 ± 0.61	1.104	0.347	-
Xenophobia		3.27 ± 1.00	3.10 ± 0.90	2.88 ± 1.00	2.37 ± 0.79	33.526	<0.001	A < C < D, B < D
Compulsivity		2.57 ± 0.84	2.72 ± 0.75	2.53 ± 0.93	2.14 ± 0.53	15.562	<0.001	A, B, C < D

A: None, B: Up to 3 months, C: 3–6 months, D: More than 6 months.

**Table 12:** Goodness-of-fit of the path model.

Model	Root Mean Square Residual	Normed Fit Index	Incremental Fit Index	Comparative Fit Index	Root Mean Square Error of Approximation
Conformity level	≤0.100	≥0.900	≥0.900	≥0.900	≤0.100
Initial model	0.079	0.905	0.918	0.918	0.080

**Table 13:** Results of the path analysis.

Hypothesis	Path			Standardized Regression Coefficient	Regression Coefficient	SE	CR	*p*	Note
H2	Participation in physical activity	→	Self-concept clarity	0.152	0.129	0.033	3.856	<0.001 ***	Accepted
H3	Participation in physical activity	→	COVID-19 stress	−0.078	−0.071	0.033	−2.152	0.031 *	Accepted
H4	Self-concept clarity	→	COVID-19 stress	−0.555	−0.602	0.050	−12.032	<0.001 ***	Accepted

SE = standard error, CR = critical ratio, * *p* < 0.05, *** *p* < 0.001, tested via path analysis.

## Data Availability

The data presented in this study are available on request to the authors.

## References

[1] Chen P., Mao L., Nassis G.P., Harmer P., Ainsworth B.E., Li F. (2020). Coronavirus disease (COVID-19): The need to maintain regular physical activity while taking precautions. J. Sport Health Sci..

[2] Laing T. (2020). The economic impact of the Coronavirus 2019 (Covid-2019): Implications for the mining industry. Extr. Ind. Soc..

[3] World Health Organization (2020). COVID-19 Weekly Epidemiological Update. https://www.who.int/publications/m/item/weekly-epidemiological-update.

[4] Pfefferbaum B., North C.S. (2020). Mental Health and the Covid-19 Pandemic. N. Engl. J. Med..

[5] Wang C., Pan R., Wan X., Tan Y., Xu L., Ho C.S., Ho R.C. (2020). Immediate Psychological Responses and Associated Factors during the Initial Stage of the 2019 Coronavirus Disease (COVID-19) Epidemic among the General Population in China. Int. J. Environ. Res. Public Health.

[6] Nearchou F., Flinn C., Niland R., Subramaniam S.S., Hennessy E. (2020). Exploring the Impact of COVID-19 on Mental Health Outcomes in Children and Adolescents: A Systematic Review. Int. J. Environ. Res. Public Health.

[7] Szlyk H.S., Berk M., Peralta A.O., Miranda R. (2020). COVID-19 Takes Adolescent Suicide Prevention to Less Charted Territory. J. Adolesc. Health.

[8] Rosenblum G.D., Lewis M., Adams G., Berzonsky M.D. (2008). Emotional Development in Adolescence. Blackwell Handbook of Adolescence.

[9] Li Y., Duan W., Chen Z. (2020). Latent profiles of the comorbidity of the symptoms for posttraumatic stress disorder and generalized anxiety disorder among children and adolescents who are susceptible to COVID-19. Child. Youth Serv. Rev..

[10] Taylor S., Landry C.A., Paluszek M.M., Fergus T.A., McKay D., Asmundson G.J. (2020). Development and initial validation of the COVID Stress Scales. J. Anxiety Disord..

[11] Selye H., Goldberger L., Breznit S. (1982). History and present status of the stress concept. Handbook of Stress: Theoretical and Clinical Aspects.

[12] Biddle S.J.H., Mutrie N. (2008). Psychological of Physical Activity: Determinants, Well-Being & Interventions.

[13] Hallal P.C., Victora C.G., Azevedo M.R., Wells J.C.K. (2006). Adolescent Physical Activity and Health. Sports Med..

[14] Heo J.H. (2019). Association between Physical Activity and Perceived Stress among Korean Adults: A Cross-Sectional Study Using 2017 the Korea National Health and Nutrition Examination Survey Data. Korean J. Stress Res..

[15] Campbell J.D. (1990). Self-esteem and clarity of the self-concept. J. Personal. Soc. Psychol..

[16] Campbell J.D., Trapnell P.D., Heine S.J., Katz I.M., Lavallee L., Lehman D.R. (1996). Self-concept clarity: Measurement, personality correlates, and cultural boundaries. J. Personal. Soc. Psychol..

[17] Treadgold R. (1999). Transcendent Vocations: Their Relationship to Stress, Depression, and Clarity of Self-Concept. J. Humanist. Psychol..

[18] Lee-Flynn S.C., Pomaki G., DeLongis A., Biesanz J.C., Puterman E. (2011). Daily Cognitive Appraisals, Daily Affect, and Long-Term Depressive Symptoms: The Role of Self-Esteem and Self-Concept Clarity in the Stress Process. Pers. Soc. Psychol. Bull..

[19] Liu M., Wu L., Ming Q. (2015). How Does Physical Activity Intervention Improve Self-Esteem and Self-Concept in Children and Adolescents? Evidence from a Meta-Analysis. PLoS ONE.

[20] Snyder E.E., Spreitzer E.E. (1983). Social Aspect of Sport.

[21] Lee S.-M., Jeong H.-C., So W.-Y., Youn H.-S. (2020). Mediating Effect of Sports Participation on the Relationship between Health Perceptions and Health Promoting Behavior in Adolescents. Int. J. Environ. Res. Public Health.

[22] Becht A.I., Nelemans S.A., Van Dijk M.P.A., Branje S.J.T., Van Lier P.A.C., Denissen J.J.A., Meeus W.H.J. (2017). Clear Self, Better Relationships: Adolescents’ Self-Concept Clarity and Relationship Quality With Parents and Peers Across 5 Years. Child Dev..

[23] Kline R.B. (2015). Principles and Practice of Structural Equation Modeling.

[24] West S.G., Finch J.F., Curran P.J., Hoyle R. (1995). Structural equation model with non-normal variables: Problems and remedies. Structural Equation Modeling: Concepts, Issues and Applications.

[25] Qi M., Li P., Moyle W., Weeks B., Jones C. (2020). Physical Activity, Health-Related Quality of Life, and Stress among the Chinese Adult Population during the COVID-19 Pandemic. Int. J. Environ. Res. Public Health.

[26] You S., Shin K. (2019). Body Esteem among Korean Adolescent Boys and Girls. Sustainability.

[27] Shevlin M., McBride O., Murphy J., Miller J.G., Hartman T.K., Levita L., Mason L., Martinez A.P., McKay R., Stocks T.V.A. (2020). Anxiety, depression, traumatic stress and COVID-19-related anxiety in the UK general population during the COVID-19 pandemic. BJPsych Open.

[28] Groffik D., Fromel K., Badura P. (2020). Composition of weekly physical activity in adolescents by level of physical activity. BMC Public Health.

[29] Shull E.R., Dowda M., Saunders R.P., McIver K., Pate R.R. (2020). Sport participation, physical activity and sedentary behavior in the transition from middle school to high school. J. Sci. Med. Sport.

[30] Rueggeberg R., Wrosch C., Miller G.E. (2012). The different roles of perceived stress in the association between older adults’ physical activity and physical health. Health Psychol..

[31] Department of Health and Human Services (2018). U.S. Physical Activity Guidelines for Americans.

[32] Van Dijk M.P., Branje S., Keijsers L., Hawk S.T., Hale W.W., Meeus W. (2014). Self-Concept Clarity Across Adolescence: Longitudinal Associations With Open Communication With Parents and Internalizing Symptoms. J. Youth Adolesc..

[33] DeMarree K.G., Rios K. (2014). Understanding the relationship between self-esteem and self-clarity: The role of desired self-esteem. J. Exp. Soc. Psychol..

[34] Streamer L., Seery M.D. (2015). Who am I? The interactive effect of early family experiences and self-esteem in predicting self-clarity. Pers. Individ. Differ..

[35] Sani S.H.Z., Fathirezaie Z., Brand S., Pühse U., Holsboer-Trachsler E., Gerber M., Talepasand S. (2016). Physical activity and self-esteem: Testing direct and indirect relationships associated with psychological and physical mechanisms. Neuropsychiatr. Dis. Treat..

[36] Vartanian L.R. (2009). When the Body Defines the Self: Self-Concept Clarity, Internalization, and Body Image. J. Soc. Clin. Psychol..

[37] Zebedee J.A., Gibbons S.L., Patti-Jean N. (2010). Family Influence on Physical Activity: Exploring the Nature of Reciprocal Relationships. Revue PhénEPS.

[38] Van Kessel G., Kavanagh M., Maher C. (2016). A Qualitative Study to Examine Feasibility and Design of an Online Social Networking Intervention to Increase Physical Activity in Teenage Girls. PLoS ONE.

[39] Carmack C.L., de Moor C., Boudreaux E., Amaral-Melendez M., Brantley P.J. (1999). Aerobic fitness and leisure physical activity as moderators of the stress-illness relation. Ann. Behav. Med..

[40] Qin F., Song Y., Nassis G.P., Zhao L., Dong Y., Zhao C., Feng Y., Zhao J. (2020). Physical Activity, Screen Time, and Emotional Well-Being during the 2019 Novel Coronavirus Outbreak in China. Int. J. Environ. Res. Public Health.

[41] Brooks S.K., Webster R.K., Smith L.E., Woodland L., Wessely S., Greenberg N., Rubin G.J. (2020). The psychological impact of quarantine and how to reduce it: Rapid review of the evidence. Lancet.

[42] Scharmer C., Martinez K., Gorrell S., Reilly E.E., Donahue J.M., Anderson D.A. (2020). Eating disorder pathology and compulsive exercise during the COVID-19 public health emergency: Examining risk associated with COVID-19 anxiety and intolerance of uncertainty. Int. J. Eat. Disord..

[43] Vartanian L.R., Dey S. (2013). Self-concept clarity, thin-ideal internalization, and appearance-related social comparison as predictors of body dissatisfaction. Body Image.

[44] O’Connor R.C., Wetherall K., Cleare S., McClelland H., Melson A.J., Niedzwiedz C.L., O’Carroll R.E., O’Connor D.B., Platt S., Scowcroft E. (2020). Mental health and well-being during the COVID-19 pandemic: Longitudinal analyses of adults in the UK COVID-19 Mental Health & Wellbeing study. Br. J. Psychiatry.

